# EVALUATION OF GRADUATES OF RESIDENCY IN DIGESTIVE SURGERY AND COLOPROCTOLOGY IN A SINGLE CENTER: A 43 YEARS PORTRAIT

**DOI:** 10.1590/0102-6720202400059e1853

**Published:** 2025-01-13

**Authors:** Gilton Marques FONSECA, Luiz Henrique DA COSTA, José Donizeti MEIRA, Nelson Fausto DELL’AQUILLA, Ulysses RIBEIRO, Paulo HERMAN, Luiz Augusto Carneiro D’ALBUQUERQUE

**Affiliations:** 1 Universidade de São Paulo, Faculty of Medicine, Departament of Gastroenterology – São Paulo (SP), Brazil.

**Keywords:** Internship and residency, Digestive system, Colorectal surgery, Gastroenterology, Internato e residência, Sistema digestório, Cirurgia colorretal, Gastroenterologia

## Abstract

**BACKGROUND:**

The medical residency model, established over a century ago, remains the gold standard for medical education. Given its increasing significance in imparting expertise in medical specialties, understanding the profile of residents and changes over time is crucial.

**AIMS:**

This study aimed to assess graduates of digestive surgery and coloproctology residency programs at Hospital das Clínicas of the Faculdade de Medicina da Universidade de São Paulo (HCFMUSP) regarding their professional, academic, and research activities. It also aimed to analyze changes in resident profiles over the years, the impact of postgraduation on professional activities, and differences between graduates working in São Paulo capital and elsewhere.

**METHODS:**

A digital survey with 42 questions was sent to graduates who commenced training between 1979 and 2021. Results were analyzed in subgroups based on two eras (Era 1: 1979–2000; Era 2: 2001–2021), the impact of postgraduation, and respondents’ work locations.

**RESULTS:**

The survey was responded by 213 graduates (87.6%). The training significantly impacted all respondents’ professional lives, with 92.5% willing to choose the same specialty again. Graduates from Era 2 showed a higher proportion of females, residents of cities other than São Paulo, and graduates from institutions outside FMUSP. Postgraduate responders were more involved in academic and research activities, publishing more papers, holding societal memberships, and performing more robot-assisted procedures. Those outside São Paulo capital were more involved in endoscopic procedures and associated with medical insurance.

**CONCLUSIONS:**

The majority of graduates considered medical residency fundamental for career development. Social and economic changes influenced residents’ profiles and post-program activities.

## RESUMO

**RACIONAL:**

O modelo de residência médica, estabelecido há mais de um século e padrão-ouro para formação dos especialistas médicos, tem crescente importância. Assim, é crucial compreender o perfil dos residentes e suas mudanças ao longo do tempo.

**OBJETIVOS:**

Avaliar os graduados da residência em Cirurgia Digestiva e Coloproctologia no Hospital das Clínicas da Faculdade de Medicina da Universidade de São Paulo (HCFMUSP) quanto às atividades profissionais, acadêmicas e de pesquisa; analisar as mudanças nos perfis dos residentes, o impacto da pós-graduação nas atividades profissionais e as diferenças entre aqueles que trabalham na capital paulista e em outras regiões.

**MÉTODOS:**

Enviado questionário digital com 42 perguntas aos graduados que iniciaram treinamento entre 1979 e 2021. Realizada análise em subgrupos conforme eras (Era 1: 1979-2000; Era 2: 2001-2021), impacto da pós-graduação e locais de trabalho.

**RESULTADOS:**

213 graduados responderam ao questionário (87,6%). O treinamento teve impacto significativo na vida profissional de todos respondentes, com 92,5% dispostos a escolher a mesma especialidade novamente. Respondedores da Era 2 apresentaram proporção maior de mulheres, residentes fora da capital paulista e formados em instituições fora da FMUSP. Aqueles pós-graduados estavam mais envolvidos em atividades acadêmicas e de pesquisa, publicando mais trabalhos, sendo membros de sociedades e realizando mais procedimentos assistidos por robôs. Aqueles fora da capital paulista estavam mais envolvidos em procedimentos endoscópicos e associados a convênio médicos.

**CONCLUSÕES:**

A maioria dos graduados considerou a residência médica fundamental para o desenvolvimento da carreira. Mudanças sociais e econômicas influenciaram os perfis dos residentes e suas atividades pós-programa.

## INTRODUCTION

The model for medical residency training originated in 1889 at Johns Hopkins University Hospital in the United States of America (USA), under the leadership of Professor William Stewart Halsted. He established a practical surgical training program post-medical school that involved long-term immersion in surgical practice, gradually increasing responsibilities until achieving final autonomy.

This program required young surgeons to dedicate themselves entirely and be available full-time for patients and surgeries, leading to them being referred to as “residents” due to their extended time spent in the hospital. The training system expanded to other medical areas, including internal medicine, pathology, and gynecology, under the leadership of Professors William Osler, William Welch, and Howard Kelly, respectively. These individuals, including William Stewart Halsted, were collectively known as “the Big Four” of Baltimore.

In 1917, the American Medical Association acknowledged the significance of medical residency, and, by 1927, the first programs received accreditation. Starting in 1933, the medical residency certificate became a prerequisite for practicing specialties in the USA^7,8,13^.

In Brazil, medical residency began in the 1940s when physicians who had trained in the USA following the Halstead model, returned to the country. The first medical residency program in orthopedics was established in 1945 at the Hospital das Clínicas of the Faculdade de Medicina da Universidade de São Paulo (HCFMUSP). Subsequently, in 1948, the Instituto de Previdência e Assistência ao Servidor do Estado do Rio de Janeiro (IPASERJ) initiated residency programs in general surgery, internal medicine, pediatrics, and gynecology^13,18^.

In the 1970s, the Brazilian government officially recognized medical residency as a postgraduate program for physicians, designating it as the “gold standard” for medical specialization in the country. To supervise and ensure quality, a National Commission was also established. As of 2021, there were 4,950 medical residency programs in Brazil, with 41,853 doctors in training, constituting approximately 8% of the active physicians in the country. Approximately half of these programs are situated in the Southeast region of Brazil, and one-third of the residents are located in São Paulo state^4,5,6,13,17,18^.

The residency program in digestive surgery was officially established in 1982 at HCFMUSP. However, starting in 1979, physicians had already been undergoing training following the same model. The residency program in coloproctology received government approval in 1981^9,11^.

In 2021, there were 243 residents in digestive surgery and 148 residents in coloproctology in Brazil, collectively constituting approximately 1% of all medical residents. The active workforce comprised 3,840 digestive surgeons in Brazil (1,606 of them in São Paulo state) and 2,414 coloproctologists (including 562 in São Paulo state) during that year. In comparison with the preceding decade, these figures reflect a notable increase of 93.3% for digestive surgeons and 67.1% for coloproctologists^17^.

Given the increasing importance of these specialties, more than 40 years since the initiation of formal training in digestive surgery, it is crucial to comprehend the evolving profile of the graduates during this period.

The objectives of the present study are to assess, among graduates of the digestive surgery and coloproctology medical residency programs at HCFMUSP:

a) their professional, academic, and research activities;
b) changes in the profile of residents over the years;
c) the impact of postgraduation on professional activities; and
d) the differences in the profile of graduates working in São Paulo capital compared to those working in other cities.

## METHODS

From April to July 2023, we conducted a survey of all graduates from the digestive surgery and coloproctology residency programs at HCFMUSP, utilizing the RedCap electronic data capture tools^12^. The inclusion criteria encompassed all living graduates in the residency program who commenced their training between 1979 and 2021. Exclusion criteria involved non-responders or graduates with unknown contact information.

The survey link was sent via email and/or instant messaging apps. The survey covered:

a) personal profile;
b) academic background;
c) surgical, academic, and research activities; and
d) other activities outside the specialty. For colleagues outside Brazil unable to access the RedCap link, we provided a Google Forms or PDF survey with identical questions.

The questionnaire was designed for brevity, allowing completion in approximately 7 minutes. We issued a total of four weekly reminders, and personal contact was established with non-responders. No incentives were provided to participants who completed the survey.

We analyzed responders in subgroups to discern three aspects: how the profile changed over the years (Era 1 – 1979 to 2000; Era 2 – 2001 to 2021), the distinction between postgraduates and non-postgraduates, and respondents working in São Paulo capital versus elsewhere. The study was approved by the Ethics Committee of the Institution (No. 69606723.0.0000.0068).

Categorical variables were presented as frequency (percentage), while continuous variables were expressed as the mean ± standard deviation and median (range). Univariate associations were assessed using the chi square test and/or Fisher’s exact test, with a p-value <0.05 considered statistically significant. Statistical analysis was conducted using Statistical Package for the Social Sciences (SPSS) for Mac, version 26 (IBM, Armonk, NY).

## RESULTS

We identified a total of 247 graduates from the digestive surgery and coloproctology residency programs at HCFMUSP who commenced training between 1979 and 2021. Four of these individuals are deceased, reducing the potential respondents to 243 graduates. Four were excluded due to unavailability of contact information, and 26 did not respond to the survey, resulting in 213 completed responses from colleagues, constituting 87.6% of potential responders. Among the responders, 19 (8.9%) completed the coloproctology program, while 194 (91.1%) completed the digestive surgery residency program. Table 1 summarizes the characteristics of the studied population.

**Table 1:** Analysis of graduates of residency in digestive surgery and coloproctology characteristics

Variable/Characteristic		n (%)
Sex		
Male		27 (12.7)
Female		186 (87.3)
Current age (years)		
Mean (SD)	46.7 (12.1)	
Median (min-max)	44.5 (27,6–74,4)	
Ethnicity		
White		177 (83.1)
Yellow		18 (8.5)
Brown		15 (7.1)
Rather not answer		3 (1.4)
Year of admission		
Era 1 (1979–2000)		80 (37.6)
Era 2 (2001–2021)		133 (62.4)
Age at admission (years)		
Mean (SD)	27.1 (1.4)	
Median (min-max)	26.9 (24,6–33.7)	
Time between graduation and admission to residency program (years)		
Mean (SD)	2.4 (0.69)	
Median (min-max)	2.0 (2–6)	
Region of origin		
Southeastern		168 (78.9)
Northeastern		19 (8.9)
South		16 (7.5)
Midwest		5 (2.3)
North		2 (0.9)
Foreigner		3 (1.4)
Origin — from São Paulo capital		
Yes		115 (54.0)
No		98 (46.0)
Origin of Medicine graduation		
FMUSP		126 (59.2)
Other public schools		61 (28.6)
Private schools		26 (12.2)
Residence of general surgery		
HCFMUSP		197 (92.5)
Other hospitals		16 (7.5)
Preceptorship		
Yes		90 (42.3)
No		123 (57.7)
Stricto sensu postgraduation		
Yes		126 (59.2)
No		87 (40.8)
Habilitation (Associate Professor)		
Yes		30 (14.1)
No		183 (85.9)
Region of the workplace		
Southeastern of Brazil		186 (87.3)
Northeastern of Brazil		9 (4.2)
South of Brazil		8 (3.8)
Midwest of Brazil		2 (0.9)
North America		5 (2.3)
Europe		2 (0.9)
Asia		1 (0.5)
Workplace in São Paulo capital		
Yes		169 (79.3)
No		44 (20.7)
Workplace in the same city of origin		
Yes		118 (55.4)
No		95 (44.6)
Institution of work		
Public and private		132 (62,0)
Only private		70 (32.8)
Only public		11 (5.2)
Academic activities		
Yes		135 (63.4)
No		78 (36.6)
Research activities		
Yes		105 (49.3)
No		108 (50.7)
Number of papers published		
Until 5		92 (43.2)
6 to 20		67 (31.5)
21 to 50		29 (13.6)
More than 50		25 (11.7)
Leadership positions		
Yes		85 (39.9)
No		128 (60.1)
Associated with medical insurance		
Yes		70 (32.9)
No		143 (67.1)
Associated with medical/ pharmaceutical companies		
Yes		13 (6.1)
No		200 (93.9)
Perform endoscopic exams/procedures		
Yes		36 (16.9)
No		177 (83.1)
Work with digestive surgery/coloproctology		
Full time		168 (78.9)
Part time		33 (15.5)
No		12 (5.6)
Perform laparoscopic procedures		
Yes		197 (92.5)
No		16 (7.5)
Perform robot-assisted procedures		
Yes		76 (35.7)
No		137 (64.3)
Specialty membership		
Yes		181 (85.0)
No		32 (15,0)

SD: standard deviation; FMUSP: Faculdade de Medicina da Universidade de São Paulo; HCFMUSP: Hospital das Clínicas da Faculdade de Medicina da Universidade de São Paulo.

Graduates originated from all regions of Brazil, representing 17 Brazilian states. Residency training had a fundamental impact on the professional life of 183 (85.9%) responders, with 30 (14.1%) reporting a great impact but not as fundamental. A total of 132 (62%) were very satisfied with the specialty, 77 (36.2%) were satisfied, and only four (1.9%) reported being somewhat satisfied. Moreover, 197 (92.5%) would certainly or probably choose the same specialty again, while only 16 (7.5%) would maybe or not choose it. Beyond the specialty, the most frequent additional activities included endoscopy (12 responders, 5.6%), management (ten responders, 4.7%), clinical specialties (eight responders, 3.7%), teaching (five responders, 2.3%), consultancy (two responders, 0.9%), pharmaceutical industry (two responders, 0.9%), retirement, audit, regulation, and forensic medical examiner (one responder each, 0.5%).

The majority of alumni work full time in digestive surgery and/or coloproctology (78.9%), with only 5.6% not working in the specialty. Almost all alumni (92.5%) perform laparoscopic procedures.

**Figure 1:**
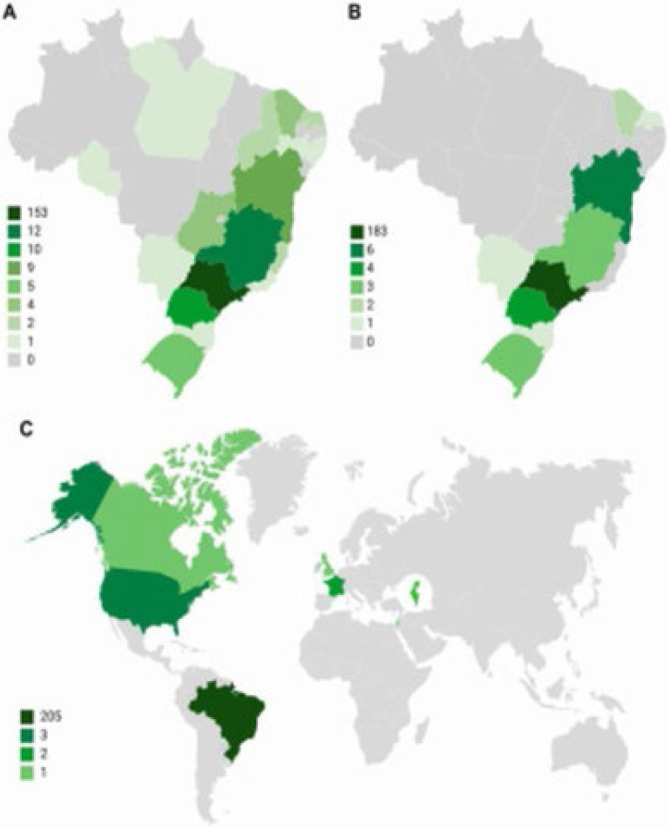
Maps depicting: A) Brazilian states of origin of graduates; B) Brazilian states of work of graduates; and C) countries of work of graduates of residency in digestive surgery and coloproctology.

Following the completion of the residency program, only a portion of the alumni returned to their region of origin. For instance, among the 19 residents from the Northeastern region, only nine (47.3%) returned, while, in the Southern region, eight out of 16 (50%) returned. Similarly, from the five residents originating from the Midwest, only two (40%) returned. Notably, none of the residents from the North returned to their home region. Figure 1 summarizes the Brazilian states from which graduates originate, as well as the Brazilian states and countries where graduates work.

Table 2 illustrates the differences between responders who underwent training in Era 1 and Era 2. In Era 2, there were more females, individuals outside São Paulo capital, and graduates outside FMUSP compared to Era 1. In Era 1, there were more postgraduates, individuals with academic title (associate professor) status, published papers, leadership positions, and specialty memberships. Additionally, responders in Era 2 graduated from medical school older (p=0.007), spent more time between graduation and specialty training (p<0.001), and, consequently, started residency training at an older age compared to Era 1 (p<0.001).

**Table 2:** Univariate analysis of differences between graduates of residency who underwent training in Era 1 (1979–2000) and Era 2 (2001–2021).

Variable	n (%)		p-value*
	Era 1 (1979–2000)	Era 2 (2001–2021)	
Sex			
Male	77 (96.2)	109 (82.0)	**0.002** ^†^
Female	3 (3.8)	24 (18.0)	
Ethnicity			
White	69 (86.3)	108 (81.2)	0.231
Yellow	8 (10.0)	10 (7.5)	
Brown	2 (2.5)	13 (9.8)	
Rather not answer	1 (1.3)	2 (1.5)	
Origin from São Paulo capital			
Yes	52 (65.0)	63 (47.4)	**0.012**
No	28 (35.0)	70 (52.6)	
Origin of Medicine graduation			
FMUSP	59 (73.8)	67 (50.4)	**0.003**
Other public schools	16 (20.0)	45 (33.8)	
Private schools	5 (6.3)	21 (15.8)	
Residence of general surgery			
HCFMUSP	77 (96.3)	120 (90.2)	0.178^†^
Other hospitals	3 (3.8)	13 (9.8)	
Stricto sensu postgraduation			
Yes	61 (76.3)	65 (48.9)	**<0.001** ^†^
No	19 (23.8)	68 (51.1)	
Habilitation (Associate Professor)			
Yes	20 (25.0)	10 (7,5)	**<0.001** ^†^
No	60 (75.0)	123 (92.5)	
Workplace in São Paulo capital			
Yes	65 (81.3)	104 (78.2)	0.594
No	15 (18.7)	29 (21.8)	
Workplace in the same city of origin			
Yes	51 (63.8)	67 (50.4)	0.065
No	29 (36.3)	66 (49.6)	
Institution of work			
Public and private	46 (57.5)	86 (64.7)	0.533
Only private	30 (37.5)	40 (30.1)	
Only public	4 (5.0)	7 (5.3)	
Academic activities			
Yes	48 (60.0)	87 (65.4)	0.464
No	32 (40.0)	46 (34.6)	
Research activities			
Yes	39 (48.8)	66 (49.6)	1.000
No	41 (51.3)	67 (50.4)	
Number of papers published			
Until 5	23 (28.8)	69 (51.9)	**<0.001**
6 to 20	23 (28.8)	44 (33.1)	
21 to 50	20 (25.0)	9 (6.8)	
More than 50	14 (17.5)	11 (8.3)	
Leadership positions			
Yes	47 (58.8)	38 (28.6)	**<0.001**
No	33 (41.3)	95 (71.4)	
Associated with medical insurance			
Yes	30 (37.5)	40 (30.1)	0.334
No	50 (62.5)	93 (69.9)	
Associated with medical/pharmaceutical companies			
Yes	5 (6.3)	8 (6.0)	1.000^†^
No	75 (93.8)	125 (94.0)	
Perform endoscopic exams/procedures			
Yes	14 (17.5)	22 (16.5)	1.000
No	66 (82.5)	111 (83.5)	
Work with digestive surgery/coloproctology			
Full time	60 (75.0)	108 (81.2)	0.504
Part time	14 (17.5)	19 (14.3)	
No	6 (7.5)	6 (4.5)	
Perform laparoscopic procedures			
Yes	72 (90.0)	125 (94.0)	0.296^†^
No	8 (10.0)	8 (6.0)	
Perform robot-assisted procedures			
Yes	30 (37.5)	46 (34.6)	0.778
No	50 (62.5)	87 (65.4)	
Specialty membership			
Yes	76 (95.0)	105 (78.9)	**<0.001** ^†^
No	4 (5.0)	28 (21.1)	

n: number of responders; FMUSP: Faculdade de Medicina da Universidade de São Paulo; HCFMUSP: Hospital das Clínicas da Faculdade de Medicina da Universidade de São Paulo. The bold values are the significant p-value (p<0.05), as stated in Methods section.

*χ^2^ test;

^†^Fisher’s exact test.

Table 3 highlights the distinctions between postgraduate and non-postgraduate responders. Postgraduate responders were more likely to be male, work in public institutions, engage in more academic and research activities, publish more papers, hold specialty memberships, and perform more robot-assisted procedures. Among the 126 postgraduate responders, 113 (89.7%) expressed a willingness to pursue postgraduate education again, while 13 (10.3%) were uncertain or would not choose it again.

**Table 3:** Distinctions between postgraduate and non-postgraduate responders.

Variable	Stricto sensu postgraduation		p-value*
	n (%)		
	Yes	No	
Sex			
Male	116 (92.1)	70 (80.5)	**0.022** ^†^
Female	10 (7.9)	17 (19.5)	
Ethnicity			
White	109 (86.5)	68 (78.2)	0.192
Yellow	10 (7.9)	8 (9.2)	
Brown	5 (4.0)	10 (11.5)	
Rather not answer	2 (1.6)	1 (1.1)	
Origin from São Paulo capital			
Yes	67 (53.2)	48 (55.2)	0.773
No	59 (46.8)	39 (44.8)	
Origin of Medicine graduation			
FMUSP	73 (57.9)	53 (60.9)	0.535
Other public schools	35 (27.8)	26 (29.9)	
Private schools	18 (14.3)	8 (9.2)	
Residence of general surgery			
HCFMUSP	119 (94.4)	78 (89.7)	0.200^†^
Other hospitals	7 (5.6)	9 (10.3)	
Workplace in São Paulo capital			
Yes	103 (81.7)	66 (75.9)	0.384
No	23 (18.3)	21 (24.1)	
Workplace in the same city of origin			
Yes	71 (56.3)	47 (54.0)	0.845
No	55 (43.7)	40 (46.0)	
Institution of work			
Public and private	92 (73.0)	40 (46.0)	**<0.001**
Only private	28 (22.2)	42 (48.3)	
Only public	6 (4.8)	5 (5.7)	
Academic activities			
Yes	101 (80.2)	34 (39.1)	**<0.001**
No	25 (19.8)	53 (60.9)	
Research activities			
Yes	87 (69.0)	18 (20.7)	**<0.001** ^†^
No	39 (31.0)	69 (79.3)	
Number of papers published			
Up to 5	30 (23.8)	62 (71.3)	**<0.001**
6 to 20	44 (34.9)	23 (26.4)	
21 to 50	28 (22.2)	1 (1.1)	
More than 50	24 (19.0)	1 (1.1)	
Leadership positions			
Yes	55 (43.7)	30 (34.5)	0.230
No	71 (56.3)	57 (65.5)	
Associated with medical insurance			
Yes	41 (32.5)	29 (33.3)	1.000
No	85 (67.5)	58 (66.7)	
Associated with medical/pharmaceutical companies			
Yes	9 (7.1)	4 (4.6)	0.566^†^
No	117 (92.9)	83 (95.4)	
Perform endoscopic exams/procedures			
Yes	23 (18.3)	13 (14.9)	0.580^†^
No	103 (81.7)	74 (85.1)	
Work with digestive surgery/coloproctology			
Full time	106 (84.1)	62 (71.3)	0.076
Part time	15 (11.9)	18 (20.7)	
No	5 (4.0)	7 (8.0)	
Perform laparoscopic procedures			
Yes	120 (95.2)	77 (88.5)	0.110^†^
No	6 (4.8)	10 (11.5)	
Perform robot-assisted procedures			
Yes	57 (45.2)	19 (21.8)	**0.001**
No	69 (54.8)	68 (78.2)	
Specialty membership			
Yes	118 (93.7)	63 (72.4)	**<0.001** ^†^
No	8 (6.3)	24 (27.6)	

n: number of responders; FMUSP: Faculdade de Medicina da Universidade de São Paulo; HCFMUSP: Hospital das Clínicas da Faculdade de Medicina da Universidade de São Paulo. The bold values are the significant p-value (p<0.05), as stated in Methods section.

*χ^2^ test;

^†^Fisher’s exact test.

Responders working in São Paulo capital more frequently originated from São Paulo capital, graduated from FMUSP, completed a residency in general surgery at HCFMUSP, had academic title (associate professor) status, engaged in research activities, and performed more robot-assisted procedures. Responders outside São Paulo capital were more involved in endoscopic procedures and more frequently associated with medical insurance (Table 4).

**Table 4:** Analysis of characteristics of graduates in residency who work and do not work in the city of São Paulo.

Variable	Workplace São Paulo capital		p-value*
	n (%)		
	Yes	No	
Sex			
Male	146 (86.4)	40 (90.9)	0.611
Female	23 (13.6)	4 (9.1)	
Ethnicity			
White	143 (84.6)	34 (77.3)	0.386
Yellow	13 (7.7)	5 (11.4)	
Brown	10 (5.9)	5 (11.4)	
Rather not answer	3 (1.8)	0 (0.0)	
Origin from São Paulo capital			
Yes	102 (60.4)	13 (29.5)	**<0.001**
No	67 (39.6)	31 (70.5)	
Origin of Medicine graduation			
FMUSP	112 (66.3)	14 (31.8)	**<0.001**
Other public schools	41 (24.3)	20 (45.5)	
Private schools	16 (9.5)	10 (22.7)	
Residence of general surgery			
HCFMUSP	160 (94.7)	37 (84.1)	**0.018**
Other hospitals	9 (5.3)	7 (15.9)	
Habilitation (Associate Professor)			
Yes	29 (17.2)	1 (2.3)	**0.011**
No	140 (82.8)	43 (97.7)	
Institution of work			
Public and private	106 (62.7)	26 (59.1)	0.851
Only private	54 (32.0)	16 (36.4)	
Only public	9 (5.3)	9 (5.3)	
Academic activities			
Yes	109 (64.5)	26 (59.1)	0.626
No	60 (35.5)	18 (40.9)	
Research activities			
Yes	91 (53.8)	14 (31.8)	**0.015**
No	78 (46.2)	30 (68.2)	
Number of papers published			
Until 5	67 (39.6)	25 (56.8)	0.076
6 to 20	54 (32.0)	13 (29.5)	
21 to 50	24 (14.2)	5 (11.4)	
More than 50	24 (14.2)	1 (2.3)	
Leadership positions			
Yes	64 (37.9)	21 (47.7)	0.309
No	105 (62.1)	23 (52.3)	
Associated with medical insurance			
Yes	46 (27.2)	24 (54.5)	0.001
No	123 (72.8)	20 (45.5)	
Associated with medical/pharmaceutical companies			
Yes	9 (5.3)	4 (9.1)	0.477^†^
No	160 (94.7)	40 (90.9)	
Perform endoscopic exams/procedures			
Yes	18 (10.7)	18 (40.9)	**<0.001**
No	151 (89.3)	26 (59.1)	
Work with digestive surgery/coloproctology			
Full time	138 (81.7)	30 (68.2)	0.146
Part time	23 (13.6)	10 (22.7)	
No	8 (4.7)	4 (9.1)	
Perform laparoscopic procedures			
Yes	157 (92.9)	40 (90.9)	0.748^†^
No	12 (7.1)	4 (9.1)	
Perform robot-assisted procedures			
Yes	67 (39.6)	9 (20.5)	**0.021** ^†^
No	102 (60.4)	35 (79.5)	
Specialty membership			
Yes	144 (85.2)	37 (84.1)	0.816^†^
No	25 (14.8)	7 (15.9)	

n: number of responders; FMUSP: Faculdade de Medicina da Universidade de São Paulo; HCFMUSP: Hospital das Clínicas da Faculdade de Medicina da Universidade de São Paulo. The bold values are the significant p-value (p<0.05), as stated in Methods section.

*χ^2^ test;

^†^Fisher’s exact test.

## DISCUSSION

The HCFMUSP, currently holding the top position in the QS University Ranking for Latin America^14^, has consistently played a pioneering role in the development of Brazilian medicine and surgery. This legacy is exemplified by the establishment of the first residency program in Brazil at this hospital, following the American model^13^.

In Brazil, the general surgery residency program had a duration of two years until 2019. Subsequently, the National Council on Medical Residency extended the duration to three years^16^. When compared to other countries, particularly in North America and Europe, where surgical programs often extend to five or more years, the duration of the general surgery residency in Brazil might be considered relatively short.

In response to this limited timeframe, it becomes crucial to provide surgeons with a more specialized field of practice through residency programs that allow for the performance of complex procedures. In this context, the residency in digestive surgery plays a pivotal role in Brazil’s surgical education, particularly as it was the first to offer such a specific program.

The specialty gained further strength with the establishment of the Brazilian College of Digestive Surgery in 1986. Its roots trace back to the 1970s when the discipline of digestive surgery at HCFMUSP was subdivided into distinct groups: esophagus, stomach, liver, pancreas and biliary tract, and colorectum. The official establishment of the residency programs in digestive surgery and coloproctology occurred in 1982 and 1981, respectively^9,11^.

After more than 40 years since the establishment of the residency program and the training of almost 250 residents at HCFMUSP, and recognizing the pivotal role that training programs play in medical and surgical education, it becomes necessary to assess the true impact of a specialty residency on the professional life of its alumni and how this impact has evolved over the years.

This study aimed to gain insights into the specialty and the residency program, paving the way for new perspectives in the future. The study revealed unanimous agreement among alumni that the residency had a significant impact on their professional lives, with 85.9% considering it fundamental for the development of their careers.

Despite the evolving landscape of medical and surgical fields, digestive surgery in Brazil remains attractive, as evidenced by 92.5% of alumni expressing their willingness to choose the same specialty again if given the chance. Moreover, a notable 94.4% continue to work part- or full-time in the specialty following the completion of their training.

It is noteworthy to observe the rising number of female residents, indicating a positive trend toward gender equality in medicine and surgery. This aligns with the broader trend seen in Brazil, where women have constituted the majority of new physicians registered with the Brazilian medical board since 2009.

Furthermore, projections suggest that, by 2024, there will be more active female doctors than male doctors^17^. Additionally, the increasing number of residents from outside São Paulo city reflects a broader trend towards decentralization of medical care. Countryside cities have taken on the responsibility of managing health care across all levels of complexity. As a result, there is a growing demand for specialized surgeons in these regions, contributing to a more widespread distribution of medical expertise and resources.

The observation that only a fraction of the residents returned to their home regions after completion of the residency program suggests that there may be perceived better work opportunities in the Southeastern region, contributing to the unequal distribution of medical and specialized care across Brazil. This phenomenon contributes to regional disparities in healthcare access and resources. The Southeast, with approximately 40% of the Brazilian population, hosts over 50% of the country’s surgeons. In contrast, the Northeast, with almost 30% of the population, has less than 20% of the country’s surgeons.

These disparities underscore the need for strategies to promote more equitable distribution of medical professionals, ensuring that various regions have access to the necessary healthcare expertise and resources^16^.

The parallel phenomenon observed in a recent survey among alumni of a hematology residency program, where the proportion of trainees from other states increased steadily while the number of hematologists practicing in other states remained stable, suggests a similar pattern^1^. It indicates that, akin to the scenario in digestive surgery and coloproctology, only a fraction of hematology residents returned to their home regions after completing their training. This pattern underscores the broader trend of uneven distribution of specialized healthcare professionals across regions, which warrants attention for more comprehensive and equitable workforce planning.

The residency program can indeed play a significant role in advancing surgical development in the country, particularly in the realm of laparoscopic surgery. The fact that almost all alumni reported performing laparoscopic surgery in their practice is noteworthy and represents a positive impact on the broader landscape of surgical techniques in Brazil^19^. This stands in contrast to the prevailing Brazilian reality, where a recent study indicates that 41.5% of cholecystectomies are still performed using open techniques in the public health system^15^.

The proficiency in laparoscopic surgery gained through these residency programs can contribute to disseminating advanced surgical methods and enhancing the overall quality of surgical care across different regions^16^. Indeed, it is plausible that alumni who pursued postgraduate studies stayed in longer contact with the institution, fostering closer ties with medical societies, contributing to more published papers, and gaining earlier exposure to newer technologies, such as robotic surgery^3^.

Postgraduate education often provides opportunities for continued learning, research, and networking, enabling individuals to stay at the forefront of advancements in their field. This could explain the observed differences in academic and research activities, publication records, and familiarity with emerging technologies between postgraduate and non-postgraduate alumni. The comparison between Era 1 and Era 2 concerning the number of postgraduates, associate professors, and leadership positions may indeed be influenced by biases. Alumni from Era 1 are older and have had more time practicing digestive surgery, contributing to a higher prevalence of these roles among them.

This pattern underscores the traditional practice in surgery where leadership positions are often held by more senior surgeons. However, the observation that almost 40% of all alumni, across both eras, occupy leadership positions in various spheres highlights the leadership vocation of the residency, extending to roles within the hospital, other public institutions, different government levels, and private practice.

Conversely, in Era 2, physicians entered the residency program at a later stage after medical graduation. One possible explanation for this delay is the need to work for a period to save resources for the residency, given that residency scholarships have not been updated for a considerable time. This financial constraint could create substantial difficulties in sustaining the cost of living in a large city like Sao Paulo, especially considering the current inflation scenario and the depreciation of the Brazilian currency.

The study does have limitations. The non-anonymous nature of the survey may have led to some responders being less candid in their responses to more subjective questions. Additionally, there could be a selection bias, as non-responders might be more dissatisfied with the institution, specialty, or residency. Furthermore, being a cross-sectional study of alumni from a single medical residency program, it may be challenging to generalize the findings to all specialties or institutions.

Indeed, our study stands out not only for being the first to specifically evaluate digestive surgeons who graduated over a period of more than 40 years from the same institution but also for achieving a high response rate of 87.6%. In the context of survey research, where response rates often hover around 60%^2^, and considering the standards set by some journals demanding rates around 80% for publishing^10^, our study surpasses these benchmarks.

This elevated response rate underscores the commitment, respect, and appreciation of the graduates for the institution, the residency program, and the specialties of digestive surgery and coloproctology. It reflects a strong connection and engagement with the alma mater, highlighting the significance of the study in shedding light on medical education and specialist training.

## CONCLUSIONS

Medical residency remains the gold standard for teaching specialties, and the majority of graduates consider it as fundamental to the development of their careers. The study also highlights the influence of social and economic changes on the profile of residents and their activities after completing the program. These insights contribute to our understanding of the evolving landscape of medical education and the ongoing impact of residency programs on the professional trajectories of graduates.
